# Pseudomorphs of barite and biogenic ZnS after phyto-crystals of calcium oxalate (whewellite) in the peat layer of a poor fen

**DOI:** 10.1007/s11356-014-2700-7

**Published:** 2014-03-08

**Authors:** Beata Smieja-Król, Janusz Janeczek, Jerzy Wiedermann

**Affiliations:** 1 Faculty of Earth Sciences, University of Silesia, Będzińska 60, 41-200 Sosnowiec, Poland; 2 Materials Properties and Structure Laboratory, Institute for Ferrous Metallurgy, 12 Miarki St., 44-100 Gliwice, Poland

**Keywords:** Ca oxalate, Metal sulfides, Barite, Authigenic minerals, Poor fen

## Abstract

Pseudomorphs of barite (BaSO_4_) and Cd-rich ZnS after whewellite (CaC_2_O_4_·H_2_O) occur within remnants of Scots pine bark tissues in the peat layer of a poor fen located near a zinc smelter in south Poland. A two-step formation of the pseudomorphs is postulated based on SEM observations: (1) complete dissolution of whewellite, possibly caused by oxalotrophic bacteria, and (2) subsequent bacterially induced precipitation of barite and spheroidal aggregates of ZnS together with galena (PbS) in voids left by the dissolved whewellite crystals. Local increase in pH due to microbial degradation of whewellite, elevated concentrations of Zn(II) and Ba(II) in pore water due to the decomposition of atmospheric particles of sphalerite and barite in the acidic (pH 3.5–3.8) environment, oxidation of S species during drying and rewetting of the peat layer, and subsequent partial reduction of sulfate anions by sulfur-reducing bacteria were all factors likely involved in the crystallization of ZnS and barite in the microenvironment of the post-whewellite voids.

## Introduction

The contamination of soil and groundwater by potentially toxic metals (e.g., Pb, Cd, Zn, and Cu) is a serious problem in areas polluted by the nonferrous industry, even if emissions today are reduced or mining and smelting activities have ceased (e.g., Li and Thornton 2001; Ek et al. 2001; Linton et al. 2007; Kierczak et al. 2013). The interaction of disseminated metals with soil components is complex and includes sorption onto organic matter and mineral surfaces, incorporation in precipitates, release through desorption and dissolution of minerals or decomposition of organic matter, and interactions with plants and microbes (Li and Thornton 2001; Helal 2006; Gadd 2010; Violante et al. 2010).

In this paper, the occurrence of barite (BaSO_4_) and unspecified polymorphs of ZnS and galena (PbS) within the space left after the dissolution of crystals of calcium oxalate monohydrate (mineral whewellite, CaC_2_O_4_·H_2_O) in a poor fen is documented. The aim is to explain the processes which lead to the precipitation of insoluble, heavy metal-bearing minerals in an environment rich in decaying organic matter. To the authors’ knowledge, this is the first report of the formation of ZnS and barite pseudomorphs after calcium oxalate. Calcium oxalate is a common constituent in soil litter, formed in large quantities by plants and fungi (Arnott 1995; Horner and Wagner 1995; Hudgins et al. 2003).

The forested poor fen of Bagno Bruch (BB) is located on the northern perimeter of the Upper Silesia industrial region in southern Poland, some 9 km east of the Zn-Pb smelter “Miasteczko Śląskie.” As revealed by ^210^Pb dating, the uppermost peat layer (∼40 cm thick) in the fen has formed over the last 200 years, i.e., during the period of intense mining and heavy industry in Upper Silesia (Smieja-Król et al. 2010). That explains the peat layer enrichment in Pb, Zn, Cd, and dust particles due to atmospheric fallout (Smieja-Król et al. 2010). Ubiquitous spherical fly ash particles and mullite in the peat layer are products of high-temperature coal combustion, whereas angular and weathered particles rich in Zn, Pb, Cd, Sn, and Sb originated in the nearby Zn-Pb smelter (Smieja-Król et al. 2010). Authigenic minerals that formed in situ in the peat layer can be distinguished from dust particles based on their morphology and spatial confinement to organic tissues. The most common of these are barite and spherical aggregates of ZnS (Smieja-Król et al. 2010).

## Materials and methods

In the summer of 2009, two peat cores (BB1 and BB2) approximately 1 m long were collected using a Wardenaar corer (Wardenaar 1986) from the thickest part (∼2 m) of the BB fen. The cores were stored at 4 °C for a few days before being cut into 1-cm slices using a stainless steel knife. A small portion of each slice (∼1 cm^3^) from the upper part of the peat profiles (0–30 cm) was fixed with 2 % glutaraldehyde for 1–2 h, then dehydrated in a graded series of ethanol (50 %, 75 %, 96 %, and 2 × 100 %, 15 min each), air-dried, mounted on aluminum specimen stubs, and sputter-coated with gold. Additionally, some samples were air-dried and, without any additional treatment, carbon-coated prior to examination by scanning electron microscopy. They were compared to the treated and gold-coated samples to exclude element redistribution and artifact formation during the fixation procedure and the gold-coating. Samples were examined using two scanning electron microscopes (SEM) each coupled to an energy-dispersive X-ray analyzer system (EDS), namely, an environmental Philips XL 30 SEM and a high-resolution FEI Inspect F SEM. Accelerating voltage of 5–25 kV and working distances between 5 and 10 mm were used to obtain EDS microanalyses, and backscattered electron (BSE) and secondary-electron (SE) images.

A 40-cm-long perforated pipe was installed approximately 1 m away from the BB coring sites to sample pore water seasonally in 2010. The water table level, temperature, and pH were measured directly during pore water sampling. Concentrations of major cations (Ca, Mg, Na, K, and Fe) were measured by atomic absorption spectrophotometry (Solaar M6), concentrations of major anions (Cl and SO_4_) were measured by using a Metrohm ion chromatograph, and concentrations of sulfide ions were obtained by the thiomercurimetric method (Wronski 1971). Trace metal concentrations (Ba, Zn, Pb, Cd) were determined by ICP-MS (PerkinElmer, ELAN 6100 DRC-e). Accuracy was found to be within 5 % as checked against the SPS-SW2 Batch 120 Surface Waters Standard.

Saturation indices of elements in pore water were calculated using a computer program, PHREEQC (Parkhurst and Appelo 2013), and the Minteq.v4 database. Input parameters used in calculations included temperature; pH; concentrations of Mg, Ca, Na, and K; and total Fe, Zn, Pb, Cd, Ba, chloride, sulfate, and sulfide.

## Results and discussion

**Fig. 1:**
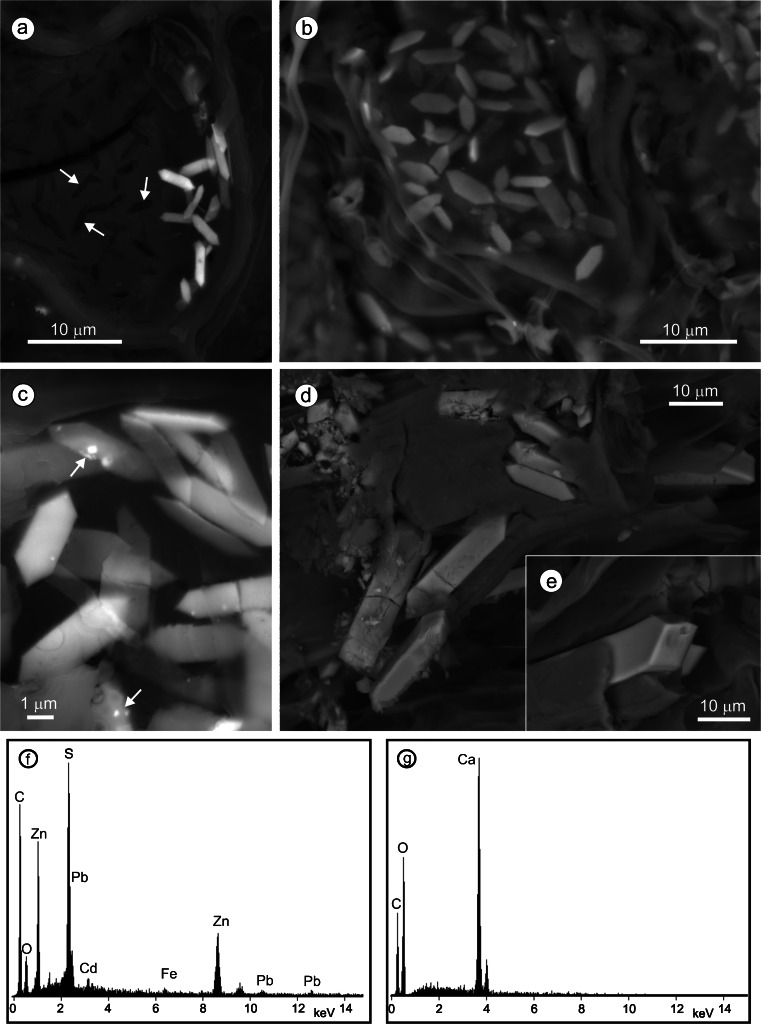
BSE images and EDS spectra of whewellite and pseudomorphs after whewellite in the peat layer of the Bagno Bruch poor fen: **a** voids (some marked by *arrows*) and barite pseudomorphs after whewellite in remnants of the Scots pine bark tissue; **b** crystals of whewellite in the bark of living Scots pine; **c** pseudomorphs of ZnS after whewellite; *arrows* point to galena inclusions; **d** and **e** whewellite crystals with well-developed {010}, {100}, {201}, and {12 $\overline{1}$} and poorly developed {110}; **f** EDS spectrum of ZnS from **c**; KPbα line is from galena; **g** EDS spectrum of whewellite shown in **b**

**Fig. 2:**
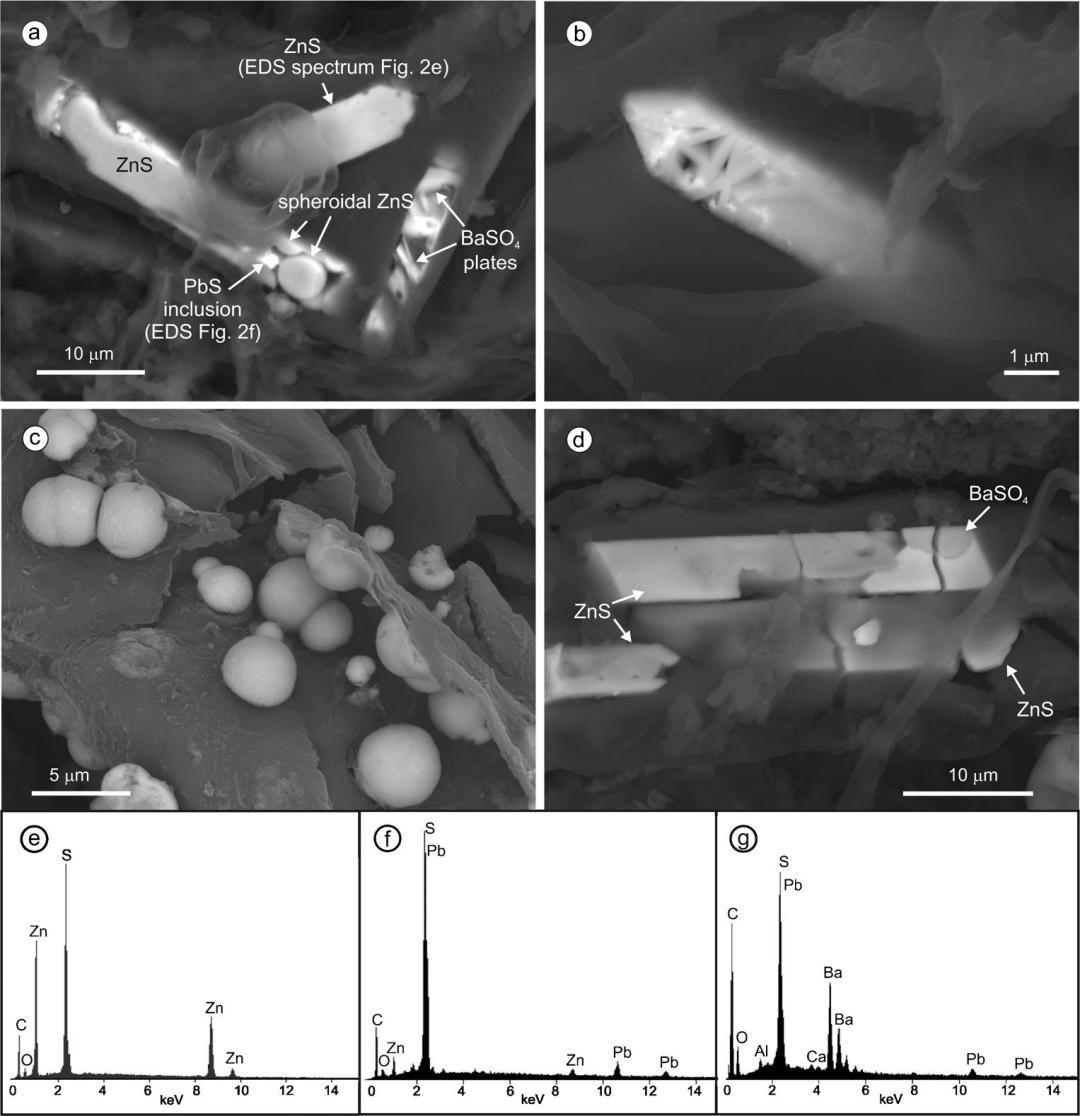
BSE images of **a** partial pseudomorphs of ZnS and barite after whewellite; **b** partial pseudomorph of barite after whewellite; the *bright spots* are galena inclusions; **c** spheroidal ZnS in remnants of plant tissues in the Bagno Bruch poor fen; **d** co-occurrence of ZnS and barite in a pseudomorph after whewellite; note the embayed grain junction between ZnS and barite; **e** EDS spectrum of ZnS from **a**; **f** EDS spectrum of galena from **a**; **g** EDS spectrum of barite with galena inclusions from **b**

Groups of doubly terminated prismatic crystals occur in remnants of Scots pine bark tissues in the peat layer of the BB fen, 16–18 cm below its surface (Figs. 1 and 2). The shapes of the crystals (<30 μm long), and their monoclinic symmetry, are identical to whewellite common in the bark of living Scots pine in the fen (Figs. 1b, d, e, g). However, microanalyses of the crystals from the peat layer reveal that they are compositionally either barite or ZnS, all with inclusions of galena (Figs. 1f and 2e, f, g). Semiquantitative EDS analyses revealed enrichment in Cd (up to ∼4 wt%) of ZnS (Fig. 1f). Low-intensity Kα lines of Al, Ca, and Pb in the EDS spectrum of the barite (Fig. 2g) reflect the presence of impurities. Lead is most probably related to galena inclusions of submicron size in the barite.

Both barite and ZnS apparently inherited their shapes from whewellite crystals, i.e., they are pseudomorphs after whewellite. Equally abundant, or even predominant, are hollow pseudomorphs after whewellite, i.e., voids left after complete dissolution of whewellite crystals (Fig. 1a). Some of these voids are partially occupied by either barite or spherical aggregates of ZnS (Figs. 2a, b, d). The latter are of the same size (a few microns in diameter) and morphology as spherical authigenic ZnS observed elsewhere in the fen (Fig. 2c). The spherical aggregates apparently represent the initial stage of ZnS growth in the vacated space. Their coalescence led to the full occupation of the available space by ZnS (Fig. 2a). Similarly, platy barite crystals grew rather randomly within the voids and gradually occupied the available space to form a complete pseudomorph after whewellite (Fig. 2b). Galena precipitated in spaces between barite plates and between spherical ZnS aggregates (Figs. 2a, b) and, as these minerals continued to grow, was encased by them.

In most cases, barite and ZnS occur separately. In rare instances, they co-occur in pseudomorphs (Fig. 2d). Spatial relationships between barite and ZnS are not entirely conclusive as to the sequence of their crystallization. However, inspection of junctions between both minerals suggests that barite crystallized first and was partially replaced by ZnS (Fig. 2d). Whereas ZnS is the dominant authigenic mineral in the fen, barite predominates over ZnS in the pseudomorphs, based on the number of crystals observed.

A two-step formation of both barite and ZnS pseudomorphs after whewellite can be inferred from SEM observations: (1) the complete dissolution of whewellite and (2) subsequent precipitation of barite and ZnS together with galena within voids formed by dissolved whewellite crystals.

The solubility of whewellite in water is low (solubility product *K*_sp_ = 2 × 10^−9^ at 25 °C; Blomen et al. 1983), and it is stable over a wide pH range (Thomas 2009). However, calcium oxalate is readily decomposed by oxalotrophic bacteria (Knutson et al. 1980; Sahin 2004; Martin et al. 2012). The bark of Scots pine that initially hosted the oxalate crystals is resistant to microbial degradation. Bark tissues are composed of highly resistant polymers including lignins and suberin and contain high concentrations of antimicrobial compounds such as terpenes, polyphenols, and stilbenes that retard decay (e.g., Pearce 1996; Berg and McClaugherty 2008). Thus, whewellite was the only bioavailable material in the bark.

The oxidation of oxalates by aerobic bacteria according to the reaction (COO^−^)_2_ + ½O_2_ + H_2_O → 2CO_2_ + 2OH^−^ results in the alkalinization of the surrounding environment (Aragno et al. 2010). For instance, the oxidation of calcium oxalate by bacteria under the iroko tree (*Milicia excelsa*) increases the soil pH from 4–6 to >8 causing the mass precipitation of calcite (Cailleau et al. 2005, 2011). The oxidation of whewellite at BB may have resulted in a local increase in pH in an otherwise acidic environment (pH 3.5–3.8; Table 1) that provided favorable conditions for the precipitation of ZnS and barite.

**Table 1:** The pore water parameters and saturation index (SI) for the top (40 cm) peat layer in the BB poor fen

	Mean	Range
Water table level (cm)	−3	−7 to 0
pH	3.6	3.5 to 3.8
Zn (μg/l)	556	381 to 720
Ba (μg/l)	300	164 to 477
Pb (μg/l)	45	23 to 110
Cd (μg/l)	2.3	1.0 to 5.5
SO_4_^2−^ (mg/l)	10.9	5.1 to 24.6
S^2−^ (mg/l)	0.71	0.3 to 1.6
pe^a^	1.0	0.9 to 1.2
SI_barite_	0.3	−0.1 to 0.8
SI_sphalerite_	1.8	1.5 to 2.2

^a^pe value calculated from the S(−II)/S(VI) redox couple

Atmospheric fallout of Fe and Zn oxides, sphalerite (ZnS), galena, Pb oxides, and barite is the primary source of Zn, Pb, and Ba ions in the BB fen (Smieja-Król et al. 2010). Zinc and Pb-rich particles were most likely emitted from the nearby zinc smelter, whereas barite related to coal combustion is a common and abundant constituent of atmospheric dust in Upper Silesia (Jablonska et al. 2001). Atmospheric dust particles characterize the top 10–18 cm of the peat layer and disappear at greater depths. This observation is consistent with chemical data that show concentrations of Zn, Pb, and Cd at 494, 238, and 16 ppm levels, respectively, in the depth interval of 12–16 cm below the surface and the concentrations of these metals decreasing to 36, 38, and 1 ppm, respectively, at a depth of 32–34 cm (Smieja-Król et al. 2010). Atmospheric particles of sphalerite and galena in the BB fen show features typical of partial dissolution, i.e., embayed grain boundaries and etched surfaces (Smieja-Król et al. 2010). The occurrence of atmospheric barite in the peat is limited to the first few centimeters below the fen surface, suggesting that barite microcrystals readily dissolve in the acidic environment of the poor fen. Despite its low solubility in water, barite is unstable in the presence of humic and fulvic acids (Smith et al. 2004). At Bagno Bruch, the dissolution of sphalerite and barite particles released Zn(II) and Ba(II) ions which subsequently precipitated as authigenic (secondary) sphalerite and barite.

The morphology, size, and internal structure of ZnS spherules in the BB fen (Fig. 2c) closely resemble micron-scale spherical aggregates of ZnS nanocrystals formed as a result of sulfate-reducing bacterial (SRB) activity (Labrenz et al. 2000; Banfield and Zhang 2001; Moreau et al. 2004; Kucha et al. 2010). The striking morphological and dimensional similarities suggest bacterially induced precipitation of ZnS in the fen. We were unable to determine which ZnS polymorph (sphalerite or wurtzite) forms the spherical ZnS aggregates. High-resolution TEM data for bacterially induced micron-scale spheroidal ZnS aggregates in a Pb-Zn mine in Wisconsin, U.S.A., showed that they consist predominantly of sphalerite nanocrystals and subordinate nanocrystals of wurtzite (Moreau et al. 2004).

There is no morphological evidence for the role of bacteria in the crystallization of barite in the barite pseudomorphs after whewellite. However, it is known that in the presence of humic substances, the crystal growth of barite is inhibited due to adsorption of these on the mineral surfaces (Smith et al. 2004). Thus, it has been suggested that barite precipitation in freshwater systems is mainly biologically mediated (Smith et al. 2004). Living organisms (protozoa) are known to precipitate euhedral barite crystals in vacuoles (Wilcock et al. 1989; Watt et al. 1991; Krejci et al. 2011). Bacterially mediated crystallization of barite under laboratory conditions has been reported and may explain the abundance of barite precipitates in microenvironments in decaying organic matter (Gonzáles-Muñoz et al. 2003).

The BB peat pore water is slightly supersaturated with respect to sphalerite and close to the saturation level for barite (Table 1). The pore water parameters showed little variation throughout the sampling period. The pore water is acidic (pH 3.5–3.8) and mildly reducing (pe 0.9–1.2). The sulfate anion (5.1–25 mg/l) predominates over the sulfide anion (0.3–1.6 mg/l). Among the metals, the highest concentrations were shown by Zn followed by Ba, Pb, and Cd (Table 1).

**Fig. 3:**
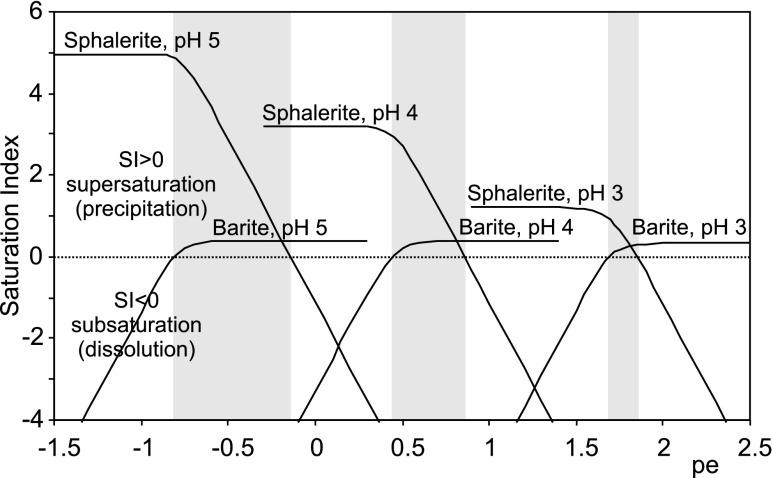
Saturation index (SI) for barite and sphalerite vs. pe at pH values of 3, 4, and 5 calculated from chemical analysis of pore water sampled in summer 2010

Both barite and ZnS polymorphs are redox sensitive. Zinc sulfide precipitates under reducing conditions, whereas barite precipitation requires more oxidizing conditions (Fig. 3), though the low-solubility field of barite extends into conditions where reduced sulfur is the predominant aqueous sulfur species (Hanor 2000). Calculations of the saturation index (SI) reveal that the stability of sphalerite is pH-sensitive (Fig. 3). An increase in pore water pH from 3 to 5 results in the increase in SI from 1.2 to 5.0. The saturation index for barite is almost pH-independent, increasing negligibly from 0.33 for pH 3 to 0.38 for pH 5. Results of geochemical calculations shown in Fig. 3 reveal the narrow range of redox conditions suitable for co-precipitation of sphalerite and barite at low pH. That range widens with increasing pH and decreasing pe (Fig. 3).

Water table fluctuation, occasionally significant at the BB fen (Smieja-Król et al. 2010), is another important factor controlling the behavior of metals and sulfur in poor fens and peat bogs. Drought-induced acidification and heavy metal release into pore water have been observed in polluted ombrotrophic peats (Tipping et al. 2003) and in other organic-rich soils (Lucassen et al. 2002; Adkinson et al. 2008). A decrease in pH weakens the strength of metal binding onto organic matter and induces metal mobilization during rewetting (Tipping et al. 2003). In the BB poor fen, an acidity drop to a pH of 3.1 and an increase in Zn concentration to 1.44 mg/l were measured in subsurface pore water close (∼1 m) to the reported ZnS and barite pseudomorphs when the fen was rewetted after a prolonged period of low water table (Smieja-Król et al. 2012). Drying and rewetting of peat due to fluctuations of the water table cause oxidation of reduced S compounds and subsequent release of sulfate ions (Eimers et al. 2003). This could explain the precipitation of barite in the BB fen and the pivotal role of SRB in reducing aqueous sulfate ion to precipitate secondary Zn and Pb sulfides there.

## Conclusions

Pseudomorphs of barite and ZnS after whewellite in the peat layer of the BB poor fen formed most probably as a result of the combination of a number of favorable conditions as follows: (1) a local increase in pH, likely caused by microbial oxidation of whewellite, elevated concentrations of Zn(II) and Ba(II) due to the dissolution of atmospheric sphalerite and barite; (2) oxidation of organic and inorganic S compounds occurred during drying and rewetting; and (3) these were subsequently partially reduced by sulfur-reducing bacteria to sulfide anions. Along with Ba and Zn, immobilization of Cd occurred through incorporation into ZnS precipitates, and Pb is retained as small inclusions of galena in barite and between ZnS microspherules. The role of calcium oxalate, a common constituent of soil litter, in modifying microsite conditions leading to mineral precipitation as suggested in this study merits further examination.
